# PCSK9 attenuates efferocytosis in endothelial cells and promotes vascular aging

**DOI:** 10.7150/thno.83914

**Published:** 2023-05-11

**Authors:** Shijie Liu, Jinzi Wu, Amanda Stolarz, Huiliang Zhang, Marjan Boerma, Stephanie D. Byrum, Nancy J Rusch, Zufeng Ding

**Affiliations:** 1 Department of Pharmacology and Toxicology, College of Medicine, University of Arkansas for Medical Sciences, Little Rock, Arkansas, USA.; 2 Department of Biomedical Informatics, College of Medicine, University of Arkansas for Medical Sciences, Little Rock, Arkansas, USA.; 3 Department of Pharmaceutical Sciences, College of Pharmacy, University of Arkansas for Medical Sciences, Little Rock, Arkansas, USA.; 4 Department of Biochemistry and Molecular Biology, College of Medicine, University of Arkansas for Medical Sciences, Little Rock, Arkansas, USA.

**Keywords:** PCSK9, endothelial cells, efferocytosis, vascular aging

## Abstract

**Aims:** Proprotein convertase subtilisin/kexin type 9 (PCSK9) is a serine protease that binds to low-density lipoprotein receptors. Efferocytosis is the process by which phagocytes remove apoptotic cells. Both PCSK9 and efferocytosis play important roles in regulating redox biology and inflammation, the key factors contributing to vascular aging. This study was designed to investigate the impact of PCSK9 on efferocytosis in endothelial cells (ECs) and its implications in vascular aging.

**Methods and Results:** Studies were performed in primary human aortic ECs (HAECs) and primary mouse aortic ECs (MAECs) isolated from male wild-type (WT) and *PCSK9^-/-^* mice, and in young and aged mice treated with saline or the PCSK9 inhibitor Pep2-8. Our findings include that recombinant PCSK9 protein induces defective efferocytosis and aging marker senescence-associated-β-galactosidase (SA-β-gal) expression in ECs, while *PCSK9^-/-^* restores efferocytosis and inhibits SA-β-gal activity. Further studies in aged mice showed that endothelial deficiency of MerTK, a critical receptor for efferocytosis that allows phagocytes to detect the presence of apoptotic cells, may be an indicator of vascular dysfunction in the aortic arch. Pep2-8 treatment markedly restored efferocytosis in endothelium from the aged mice. A proteomics study in the aortic arch from aged mice revealed that Pep2-8 administration significantly downregulates expression of NOX4, MAPK subunits, NF-κB, and secretion of pro-inflammatory cytokines, all known to promote vascular aging. Immunofluorescent staining showed that Pep2-8 administration upregulates expression of eNOS and downregulates expression of pro-IL-1β, NF-κB and p22^phox^ compared to saline treated group.

**Conclusions:** These findings provide initial evidence for the ability of aortic ECs to accomplish efferocytosis and argue for a role of PCSK9 in attenuating EC efferocytosis, thereby leading to vascular dysfunction and acceleration in vascular aging.

## Introduction

The senescence of vascular endothelial cells (ECs) and subsequent vascular aging have been suggested as key mechanisms underlying aging-related diseases.^1^ Oxidative stress is known to be an important contributor to EC senescence.^2^ There are a variety of reactive oxygen species (ROS) within ECs, including hydrogen peroxide, peroxynitrite, and hydroxyl radical.^3^ Nicotinamide adenine dinucleotide phosphate (NADPH) oxidase-mediated cellular ROS is one of the main sources of ROS production in ECs, and ROS-induced cellular stress and injury are driving factors to promote EC senescence.^2^,^3^ Cytokine-mediated inflammation is recognized as another key factor in the induction of EC senescence.^4^ Pro-inflammatory cytokines with a known role in EC senescence include interleukin (IL)-1β, IL-18, IL-6, and tumor necrosis factor-alpha (TNF-α).^5^ The pro-inflammatory response is related to activation of nuclear factor-kappa B (NF-κB) signaling, which promotes inflammatory cytokine expression during EC senescence.^2^

Proprotein convertase subtilisin/kexin type 9 (PCSK9) plays a regulatory role in cholesterol homeostasis by promoting degradation of low-density lipoprotein receptors (LDLr).^6^ PCSK9 is mainly secreted by the liver and kidney, but recent studies show that PCSK9 also is expressed in vascular cells, such as ECs and smooth muscle cells (SMCs).^7^,^8^ PCSK9 can directly induce ROS production, upregulate the expression of pro-inflammatory cytokines (e.g., IL-1β, IL-18, IL-6, TNF-α) and NF-κB.^9^ Although all these processes are involved in the development of EC senescence^10^, the relationship between PCSK9 and EC senescence apparently has not been explored.

Apoptosis is a programmed cell death and plays an important role in the vascular development and homeostasis.^11^ Moreover, efferocytosis, the process by which phagocytes clear apoptotic cells, plays a key role in resolving inflammation.^12^ Oxidative stress drives defective efferocytosis, leading to secretion of pro-inflammatory cytokines such as IL-1β and TNF-α.^13^ Current evidence indicates that both defective efferocytosis and programmed cell death are involved in promoting EC senescence.^14^,^15^

We recently reported that PCSK9 promotes ROS production, induces mitochondrial DNA damage, and activates the NLRP3 inflammasome, all of which are known to promote defective efferocytosis.^7^,^8^,^13^,^16^-^18^ Therefore, we hypothesize that PCSK9 may play a role in EC senescence by impairing efferocytosis. Our studies considered involvement of c-Mer tyrosine kinase (MerTK), an efferocytosis receptor,^17^ in the relationship between PCSK9 and EC efferocytosis in vascular aging.

## Methods

**Animals.** Male wild-type (WT) and *PCSK9^-/-^* mice on the C57BL/6 background were purchased from Jackson Laboratories (Sacramento, CA, USA) and housed in the Division of Laboratory Animal Medicine at our institution. All experimental procedures were performed in accordance with protocols approved by the Institutional Animal Care and Use Committee and conformed to the Guidelines for the Care and Use of Laboratory Animals published by the US National Institutes of Health. To avoid the effect of sex on PCSK9 levels, only male mice at the age of 3 (young) or 24 months (aged) were used in this study. To study the effect of PCSK9 on vascular aging, *PCSK9^-/-^* mice or aging WT mice were subcutaneously administered the PCSK9 inhibitor Pep2-8 (Sigma SML1132, 10 µg/kg body weight in 100 µL saline) or 100 µL saline every 2 weeks for 10 weeks before reaching the age of 24 months (n=10/group). All procedures that may cause pain or distress to the mice were done according to UAMS IACUC-approved SOPs under general anesthesia isoflurane (2.5% induction and 1.5% maintenance at 1 L/min oxygen). Mice were monitored every day for 1 week after Pep2-8 injection.

**Tissue collection.** After mice were euthanized by CO_2_ asphyxiation at the 3-month or 24-month endpoint, the aortic tree from the aortic root to thoracic aorta was carefully dissected from surrounding tissue, then fixed with 10% neutral buffered formalin solution (Sigma, HT501128) and embedded in paraffin, or stored at -80°C for further molecular, histological and immunohistochemical analyses.

**Cells.** Primary mouse aortic ECs (MAECs) were isolated from WT and *PCSK9^-/-^* mice according to the protocol provided by Wang et al.^19^ Primary human aortic ECs (HAECs) and the human Jurkat cell line were purchased from ATCC (Manassas, VA, USA). MAECs and HAECs were cultured with Vascular Cell Basal Medium (ATCC, PCS-100-030) and Endothelial Cell Growth Kit (ATCC, PCS-100-041). Jurkat cells were cultured in RPMI-1640 medium (ATCC, 30-2001) with 10% Fetal Bovine Serum (FBS, ATCC 30-2020). Recombinant human PCSK9 protein (Abcam, ab198471) and recombinant mouse PCSK9 protein (Abcam, ab167759) at indicated concentrations were used to study the role of PCSK9 in regulating MerTK expression in MAECs and HAECs. Passage 3 (P3) and Passage 15 (P15) ECs were used as young and aged cells, respectively.

**Efferocytosis evaluation.** EC efferocytosis was analyzed by a protocol modified from a published method.^17^,^18^ Briefly, ECs were plated (1 × 10^6^ cells/well) on a 6-well cell culture plate and allowed to reach confluence. Jurkat cells, an acute T-cell leukemia cell line widely used for efferocytosis studies in cardiovascular disease^47^-^49^, were labeled with PKH67-GL (2 μM; Sigma-Aldrich) and exposed to UV light (254 nm, UVP) for 5 min to induce apoptosis; they then were incubated at 37 °C with 5% CO_2_ for 1 h. EC medium was replaced with medium containing apoptotic Jurkat cells to achieve a cell ratio of 3:1, or as indicated for apoptotic Jurkat cells/ECs. After incubation for 1 h at 37 °C, the ECs were washed twice with cold PBS. The percentage of ECs labeled with PKH67-GL from engulfing apoptotic cells were quantified with fluorescence microscopy or flow cytometry.

**Western blot.** Protein was extracted with RIPA Lysis Buffer System (Santa Cruz, CA, USA) and loaded onto Mini-PROTEAN® TGX™ Precast Gels (Bio-rad, CA, USA) for electrophoresis. The size-separated proteins were then transferred to Hybond ECL Nitrocellulose Membranes (GE Healthcare, NJ, USA). After blocking with 5% BSA buffer for 1 h, the membranes were incubated with primary antibody recognizing either PCSK9 (Abcam, ab31762), MerTK (Santa Cruz, sc-365499), eNOS (Cell Signaling, 32027S), NLRP3 (Cell Signaling, 15101S), IL-1β (Cell Signaling, 12703S), NF-κB (Cell Signaling, 8242S), TNF-α (Cell Signaling, 3707S), p22^phox^ (Cell Signaling, 37570S), or β-actin (Abcam, ab227387) at 1:1000 dilution overnight at 4°C. After washing with PBS containing 0.1% Tween-20, membranes were incubated with secondary antibody targeting either anti-rabbit (Abcam, ab6721) or anti-mouse (Abcam, ab6708) at 1:4000 dilution for 1 h and signals were detected with Pierce ECL western blotting substrate (Thermo Fisher Scientific, MA, USA). Intensity quantification of the bands was performed with Image J software and normalized to β-actin.

**Measurement of cellular ROS by flow cytometry.** Cellular total ROS generation was measured by flow cytometry (BD LSRFortessa) with a Cellular ROS Assay Kit (Abcam, ab186027) according to the provided protocol. Data were analyzed by FlowJo 7.6.1.

**β-Galactosidase Staining.** *In vitro*, cell senescence in ECs was performed with a Senescence β-Galactosidase Staining Kit (Cell Signaling, 9860. *In vivo*, cell senescence in the aorta was examined with a Senescence Detection Kit (Abcam, ab65351) according to the provided protocol.

**Immunohistochemical and immunofluorescent staining.** For immunohistochemical analyses, 5 µm thick sections of the aorta were stained with indicated antibodies and analyzed using Mouse/Rabbit Specific HRP/3,3'-diaminobenzidine detection immunohistochemistry kit (Abcam, ab64264) according to the provided protocol. Immunofluorescent staining was performed by Immunofluorescent Staining of Paraffin-Embedded Tissue provided by Novus Biologicals. The information of antibodies is shown as follows: Tie2 antibody (Thermo Fisher, 14-2029-82), MerTK antibody (Abcam, ab300136), eNOS (Cell Signaling, 32027S), IL-1β (Cell Signaling, 31202S), NF-κB (Cell Signaling, 8242S) and p22^phox^ (Cell Signaling, 37570S).

**ELISA analysis.** PCSK9 concentration in serum from both young (3-month-old) and aged (24-month-old) mice (n=10) was analyzed by Biolegend LEGEND MAX™ Mouse PCSK9 ELISA Kit (Fisher Scientific, 50-207-9999) according to the provided protocol.

**RNA sequencing (RNA-seq) in HAECs.** RNA was extracted from 5 randomly selected frozen specimens of aorta per experimental group using Quick DNA/RNA miniprep kit (Zymo Research, CA, USA). The quality of RNA was assessed using Qubit RNA Broad Range Assay and fragment analyzer (Agilent, CA, USA). RNA libraries were prepared using TruSeq Stranded Total RNA Kit (Illumina, CA, USA) and then sequenced on a NovaSeq 6000 system with a SP 200-cycle flow cell. RNA-seq reads were quality-checked, trimmed, and aligned to the GRCm39 reference genome (accession: GCA_000001635.9) using the Nextflow RNAseq pipeline, nf-core/rnaseq (version 3.4 available at DOI 10.5281/zenodo.1400710). The resulting gene counts were transformed to log2 counts per million (CPM).^20^ Genes with a low expression were filtered out and libraries were normalized by trimmed mean of M-values.^21^ The Limma R package was used to calculate differential expression among genes.^22^ Log2 fold change values were calculated for each sample compared to control. Genes with an absolute fold change > 2 were considered significant.

**Proteomics measurements in the aortic arch.** Proteomics in the aortic arch from 24-month-old WT mice treated with PCSK9 inhibitor Pep2-8 or saline every 2 weeks for 10 weeks before reaching the age of 24 months (n=5/group), was performed with Orbitrap Exploris 480 Mass Spectrometer (Thermo) at the IDeA National Resource for Quantitative Proteomics at our institution using a data independent acquisition (DIA) protocol. Following data acquisition, data were searched using an empirically corrected library and a quantitative analysis was performed to obtain a comprehensive proteomic profile. Proteins were identified and quantified using EncyclopeDIA^23^ and visualized with Scaffold DIA using 1% false discovery thresholds at both the protein and peptide level. Protein MS2 exclusive intensity values were assessed for quality using ProteiNorm.^24^ The data were normalized by cyclic loss in order to perform statistical analysis using linear models for microarray data (limma) with empirical Bayes (eBayes) smoothing to the standard errors.^25^ Proteins with an FDR adjusted p-value < 0.05 and a fold change > 2 were considered significant. The proteomics data were analyzed by Ingenuity Pathway Analysis (IPA) software (Qiagen).

**Statistical analysis.** Statistical analysis was performed with GraphPad Prism 9.0. Data were summarized as the mean ± standard deviation (SD). An unpaired Student's *t*-test was used to determine statistical significance. Comparisons between multiple points were subjected to two-way ANOVA with Tukey's or Dunnett's post hoc tests; *P* < 0 .05 was considered significant.

## Results

### PCSK9 and EC efferocytosis

**Figure 1:**
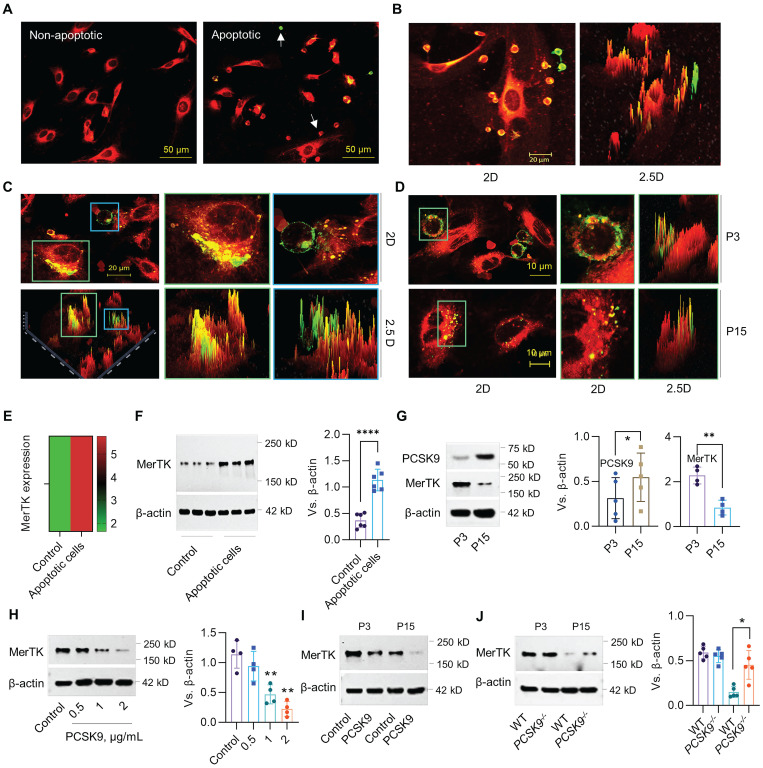
** Evidence for EC efferocytosis and PCSK9 regulation of MerTK expression.** (**A**) Uptake of normal or apoptotic Jurkat cells by human aortic endothelial cells (HAECs, P3) after 1 hour of co-incubation. (**B**) Efferocytosis of apoptotic Jurkat cells by P3 HAECs after 1 hour of co-incubation. (**C**) Confocal microscopy at 63x magnification verifies efferocytosis by P3 HAECs. (**D**) Efferocytosis in P3 and P15 HAECs. (**A-D**) Jurkat cells were labeled with green PKH67-GL (2 μM; Sigma-Aldrich) and HAECs were labeled with red PKH67-GL (2 μM; Sigma-Aldrich) per the manufacturer's instructions. Green cells are apoptotic Jurkat cells that were not engulfed by HAECs. Green/red small round cells are apoptotic Jurkat cells that were engulfed by HAECs. Large red cells are HAECs. (**E-F**) RNA-seq or western blot for MerTK expression in HAECs. (**G**) Expression of PCSK9 and MerTK in young (P3) and aged (P15) HAECs. (**H**) Treatment with PCSK9 or control PBS for 24 hours at indicated concentrations and MerTK expression in young P3 HAECs. (**I**) MerTK expression in young and aged HAECs treated with PBS (control) or PCSK9 at 1 µg/mL for 24 hours. (**J**) MerTK expression in P3 and P15 primary mouse aortic endothelial cells (MAECs) isolated from WT and *PCSK9^-/-^* mice. Statistical analyses were performed with GraphPad Prism 9.0 using a two-tailed unpaired t-test. Data represent mean ± SD (n=4-6). *P< 0.05, **P<0.01, ****P< 0.0001.

The phagocytic clearance of apoptotic cells called efferocytosis is mainly performed by macrophages.^26^,^27^ However a recent study showed that MerTK, an efferocytosis receptor generally associated with macrophages, is highly expressed in ECs, indicating the potential of ECs to perform efferocytosis.^28^ Human Jurkat cells (~10.72 μm in diameter), an acute T cell leukemia cell line, have been widely used for efferocytosis studies in cardiovascular disease (CVD).^17^,^18^ Our studies indicate that primary HAECs (passage 3, P3) prefer to uptake apoptotic Jurkat cells compared to normal Jurkat cells (**Figure 1A**). More interestingly, P3 HAECs have a high ability to engulf apoptotic Jurkat cells, indicating robust efferocytosis activity in these ECs (**Figure 1B**). Subsequently, HAECs can degrade apoptotic Jurkat cells within an hour (**Figure 1C**). Further studies with both young (passage 3, P3) and aged (passage 15, P15) HAECs showed that aged HAECs mainly uptake cell debris instead of intact apoptotic cells (**Figure 1D**). For MerTK detection in ECs, our data from both bulk RNA sequencing (RNA-seq) and western blot showed that apoptotic cells induce MerTK expression in HAECs (**Figure 1E** and **1F**). We also observed that PCSK9 expression increased whereas MerTK expression decreased in aged ECs compared with young ECs (**Figure 1G**). The addition of human recombinant PCSK9 protein at 1 and 2 μg/mL for 24 h significantly decreased MerTK expression in young HAECs (**Figure 1H**). We further confirmed that PCSK9 at 1 μg/mL inhibited MerTK expression in both young and aged cells (**Figure 1I**). Consistently, our findings in primary MAECs isolated from WT and *PCSK9^-/-^* mice confirmed that PCSK9 gene deletion partially restores MerTK expression in aged ECs (**Figure 1J**). These findings provide persuasive evidence for the high ability of young ECs to accomplish efferocytosis and argue for a role of PCSK9 in attenuating EC efferocytosis, prompting us to expand our focus to include the effect of PCSK9 inhibition on vascular aging.

### PCSK9 inhibitor treatment and EC efferocytosis

**Figure 2:**
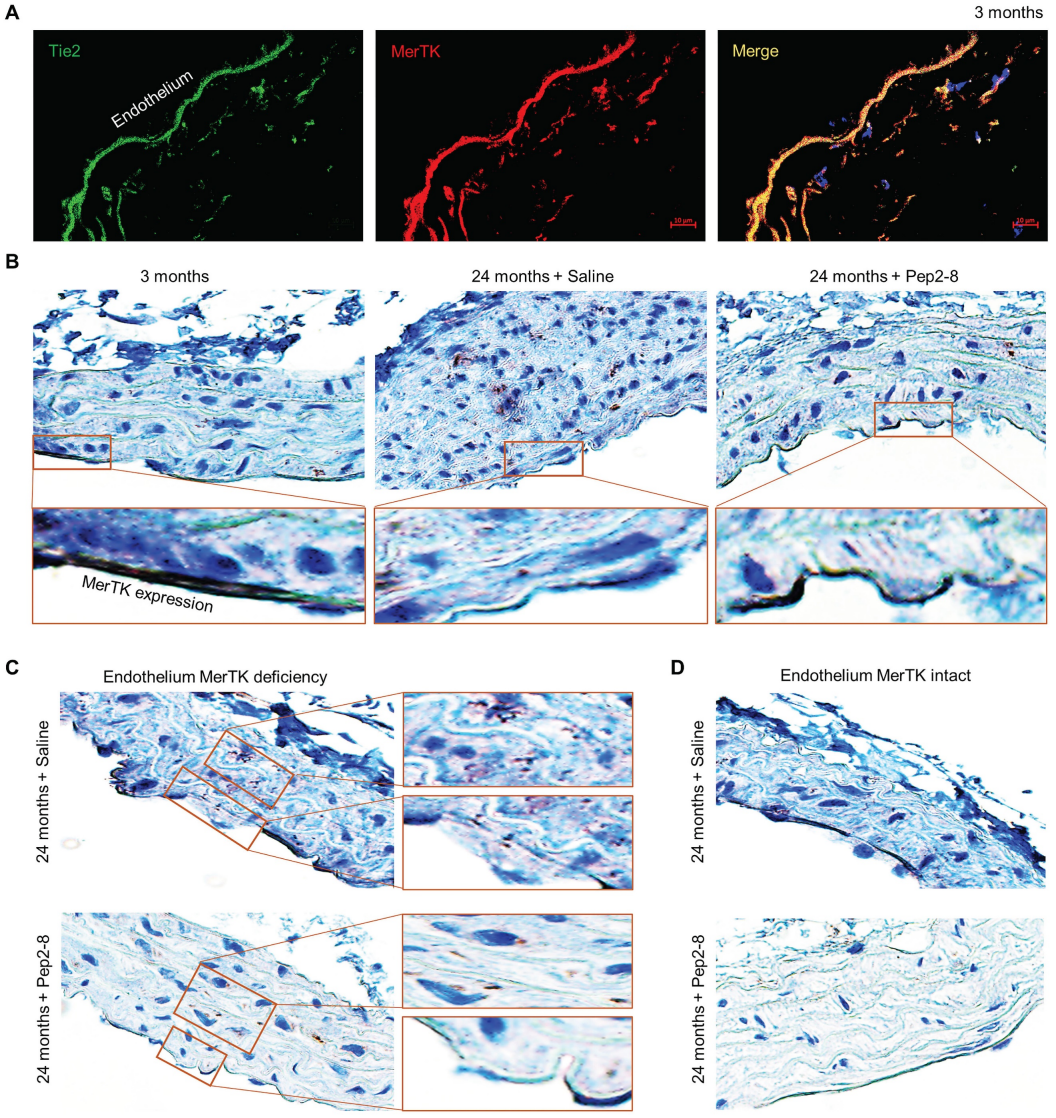
** Loss of endothelial MerTK expression in aged aortic arch is restored by PCSK9 inhibition.** (**A**) Immunofluorescence staining for expression of Tie2 and MerTK in the aortic arch of young (3-month-old) mice in paraffin sections. (**B**) Immunohistochemical staining for MerTK expression in aortic arch from young (3-month-old) mice, and aged (24-month-old) mice injected with saline or PCSK9 inhibitor Pep2-8. (**C-D**) Deficient expression of MerTK in aortic endothelium of aged mice is associated with increased efferocytosis in the underyling medial layer. The mice were subcutaneously administered Pep2-8 (10 µg/kg body weight in 100 µL saline) or 100 µL saline every 2 weeks for 10 weeks before reaching the age of 24 months (n=10/group).

Two PCSK9 inhibitors, alirocumab and evolocumab, have been approved by the U.S. Federal Drug Administration (FDA) for treating dyslipidemias.^29^ We believe that repurposing of a PCSK9 inhibitor may attenuate vascular senescence while also verifying the contribution of PCSK9 to vascular aging. Pep2-8 is a potent PCSK9 inhibitor that selectively binds to PCSK9 and interferes with LDL receptor binding to PCSK9.^30^ Pep2-8 restores LDL receptor function and LDL uptake of PCSK9-treated HepG2 cells.^30^ We administered Pep2-8 (10 µg/kg body weight in 100 µL saline, or 100 µL saline as control) subcutaneously every 2 weeks for 10 weeks to WT mice until they reached the age of 24 months. Then we evaluated MerTK expression in the aortic arch, an area that is prone to endothelial dysfunction.^31^ Findings were compared to the aortic arch of 3-month-old male WT mice. Our immunofluorescence staining with Tie2, a receptor tyrosine kinase expressed predominantly in ECs,^32^ and MerTK showed that MerTK is mainly expressed in endothelium of 3-month-old mice (**Figure 2A**). Immunohistochemical staining confirmed that MerTK is indeed highly and mainly expressed in endothelium in the young aortic arch. MerTK expression decreased while Pep2-8 restored MerTK expression in endothelium of the aortic arch from 24-month-old mice (**Figure 2B**), implying that PCSK9 inhibition is benefical for endothelial efferocytosis in the aged aorta. Interestingly, we found that endothelium areas lacking MerTK appeared to exhibit increased efferocytosis possibly in macrophages (brown dots) in the arterial media, an indicator of increased apoptotic cells that is associated with vascular dysfunction (**Figure 2C**), whereas efferocytosis was minimal in media near endothelium areas with intact MerTK (**Figure 2D**). These findings raise the intriguing possibility that endothelial MerTK-mediated efferocytosis may play a key protective role to mitigate endothelial aging and other cellular processes contributing to atherosclerosis.

### PCSK9 and vascular aging

**Figure 3:**
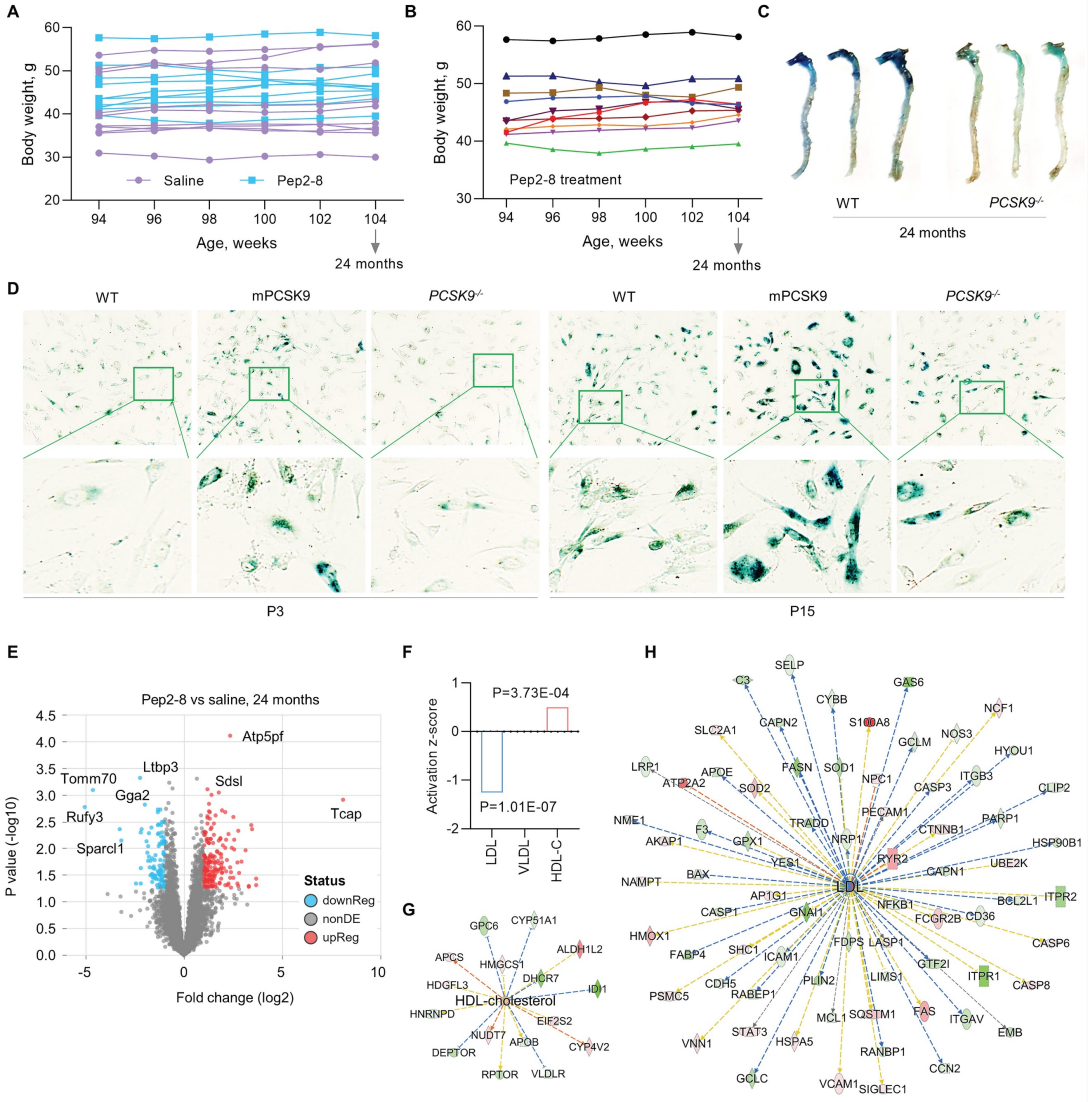
** Pep2-8 treatment postpones the aging process.** (**A**) Bodyweight of Pep2-8 or saline treated WT mice. (**B**) More detailed bodyweight from Pep2-8 treated group based on (**A**) in aged WT mice. (**C**) β-Galactosidase staining as a senescence marker in the whole aorta from WT and *PCSK9^-/-^* 24-month-old mice. (**D**) β-Galactosidase staining in P3 (left six panels) and P15 (right six panels) MAECs isolated from WT and *PCSK9^-/-^* mice. WT MAECs were treated with mouse recombinant PCSK9 at 0.5 µg/mL for 1 week. (**E**) Volcano plot illustrating differentially expressed proteins in the aortic arch from Pep2-8 or saline treated aged WT mice. Relative protein abundance (log2) plotted against significance level (- log10 P-value), showing significantly (*p* < 0.05) downregulated (blue), upregulated (red) or non-differentially expressed proteins (gray). (**F**) Pep2-8 regulates LDL and HDL-cholesterol (HDL-C), but not VLDL, based on activation z-score. (**G-H**) IPA prediction of HDL-C and LDL networks. Upregulated and downregulated proteins are highlighted in red and green, respectively, and the color depth is correlated to the fold change. Orange and blue dashed lines with arrows indicate indirect activation and inhibition, respectively. Yellow and gray dashed lines with arrows depict inconsistent effects and no prediction, respectively.

PCSK9 increases ROS production, the inflammatory response, and programmed cell death, all key factors in aging,^33^ implicating PCSK9 as a potentially important contributor to vascular aging. To investigate the role of PCSK9 in regulating vascular aging, Pep2-8 or saline was subcutaneously administered to mice every 2 weeks for 10 weeks before reaching the age of 24 months. The body weights of saline and Pep2-8 treated mice are shown in **Figure 3A-B.** Pep2-8 treatment had no effect on body weight in aged mice. One of the features of cellular senescence is increased activity of senescence-associated-β-galactosidase (SA-β-gal).^34^ Our data showed that SA-β-gal activity is markedly decreased in aorta of aged (24-month-old) *PCSK9^-/-^* mice compared to aorta of age matched WT mice (**Figure 3C**). Similarly, SA-β-gal staining of aged MAECs revealed that *PCSK9^-/-^* markedly decreased SA-β-gal activity compared to WT MAECs (**Figure 3D**). In contrast, treatment of aged MAECs with recombinant mouse PCSK9 protein (mPCSK9) aggravated SA-β-gal activity in both young and aged MAECs particularly in small areas (**Figure 3D**).

PCSK9 inhibitors are highly efficacious lipid lowering drugs.^35^ To investigate whether Pep2-8 plays a role in regulating lipids in the aged mice, proteomics analysis of the aortic arch was performed. As shown in **Figure 3E**, there was a clear separation in protein profiles between Pep2-8 and saline treated groups. A total of 293 proteins including 197 upregulated proteins and 96 downregulated proteins in the Pep2-8 treated group were identified. These proteomics data show that Pep2-8 significantly decreases LDL and increases high density lipoprotein (HDL) compared with saline treatment (**Figure 3F**). On the other hand, Pep2-8 treatment had no effect on very low-density lipoproteins (VLDL) (**Figure 3F**). Ingenuity Pathway Analysis (IPA) was performed to investigate the most enriched biological functions and molecular networks of PCSK9 targets in the aged aortic arch. As shown in **Figure 3G** and **3H**, IPA prediction revealed HDL and LDL related signaling networks, suggesting the involvement of proteins such as Glypican 6 (GPC6, a member of a family of glycosylphosphatidylinositol-anchored heparan sulfate proteoglycans), Lysine-Specific Demethylase 1-like histone demethylase (LDL1), and growth-arrest specific 6 (GAS6, another key factor for efferocytosis) in PCSK9-mediated lipid degradation.

### Mechanism of PCSK9-mediated defective efferocytosis

Efferocytosis is a process to clear apoptotic cells while maintaining homeostasis, preventing autoimmune disease and promoting resolution of inflammation.^26^ However, a variety of factors, such as excessive production of ROS or pro-inflammatory cytokines, lipopolysaccharide (LPS) and oxidized low-density lipoprotein (Ox-LDL), inhibit MerTK expression and induce MerTK cleavage, leading to defective efferocytosis.^13^ It is noted that ROS production and chronic inflammation (inflammaging) play critical roles in aging and age-related diseases.^36^ Therefore, we explored the contributions of ROS and pro-inflammatory pathways to PCSK9-mediated vascular aging.

**Figure 4:**
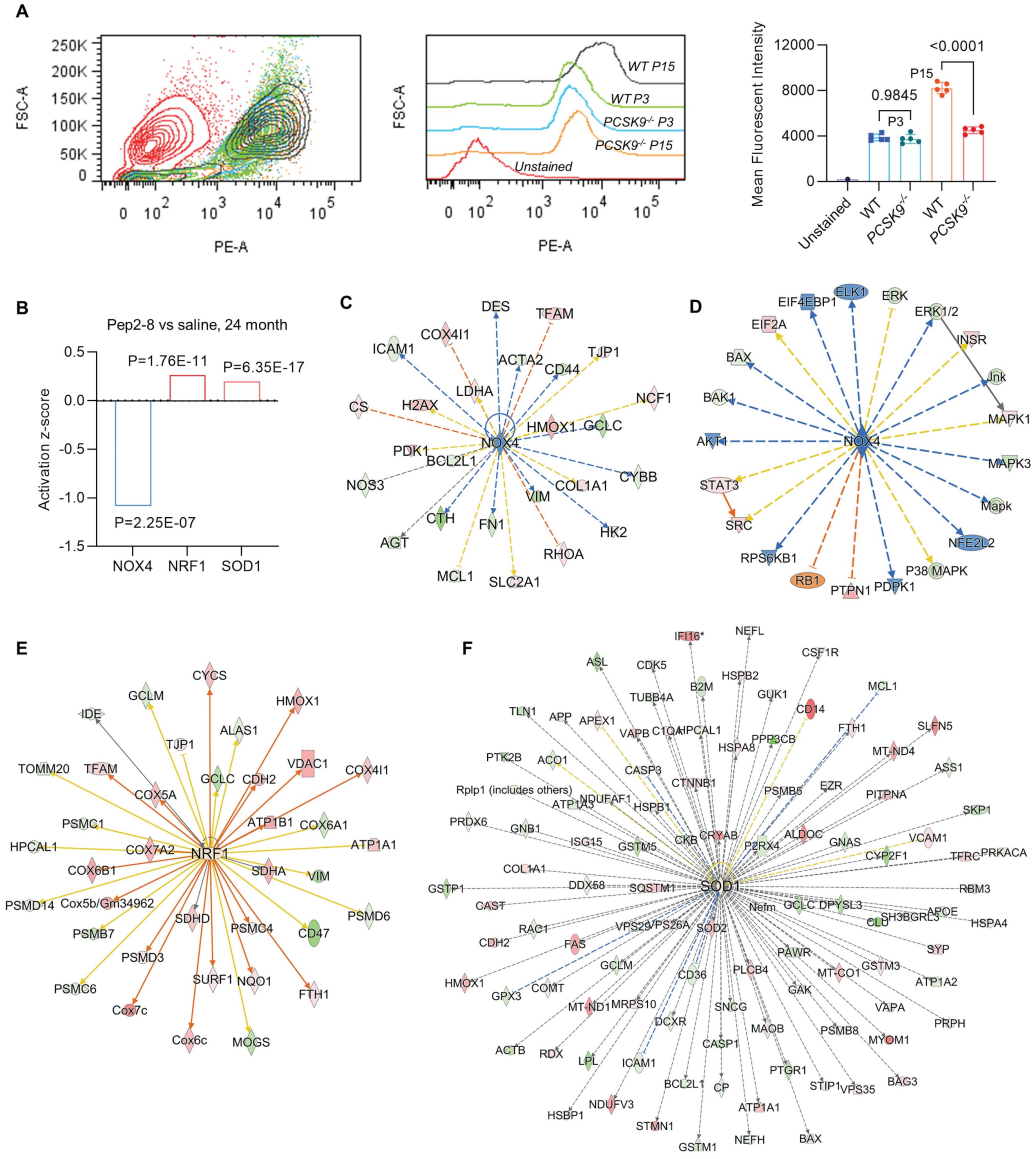
** PCSK9 inhibition and ROS production.** (**A**) Flow cytometry analysis of ROS production in young (P3) and aged (P15) MAECs isolated from WT and *PCSK9^-/-^* mice. Statistical analyses were performed with GraphPad Prism 9.0 using a two-tailed unpaired t-test. Data represent mean ± SD (n=5). (**B**) Pep2-8 treatment and activation z-score of NOX4, NRF1 and SOD1. (**C-F**) IPA prediction and (**D**) causal networks of NOX4, NRF1 and SOD1. Upregulated and downregulated proteins are highlighted in red and green, respectively, and the color depth is correlated to the fold change. Orange and blue dashed lines with arrows indicate indirect activation and inhibition, respectively. Yellow and gray dashed lines with arrows depict inconsistent effects and no prediction, respectively.

**Figure 5:**
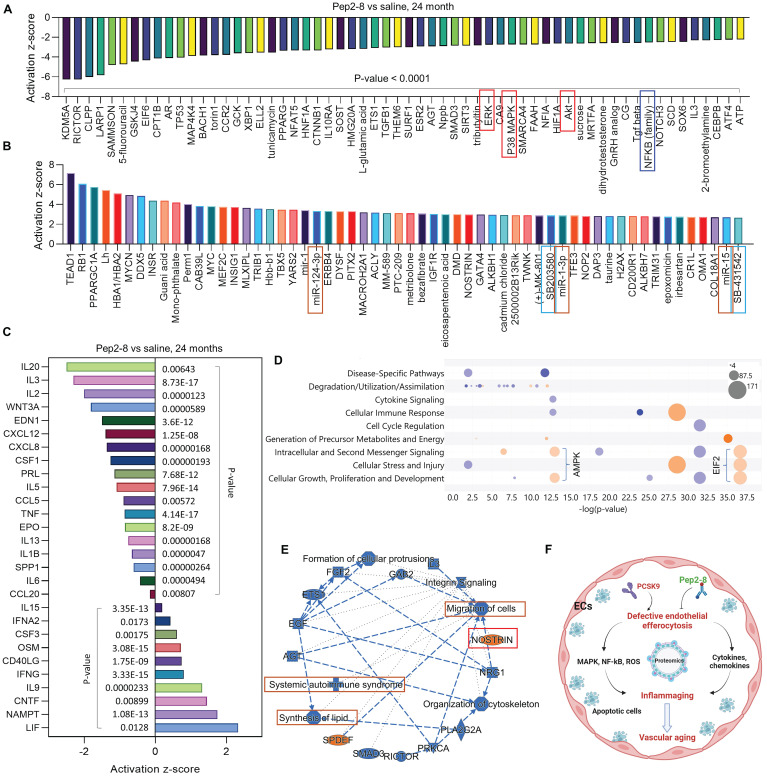
** Pep2-8 treatment and protein changes** in the aortic arch of WT 24-month-old mice injected with Pep2-8 vs saline every two weeks for 10 weeks**.** (**A**-**B**) 60 representative proteins downregulated or upregulated by Pep2-8 in aged WT mice based on activation z-score. (**C**) Pep2-8 treatment regulates inflammatory cytokines or chemokines based on activation score in the aortic arch of 24-month-old mice. (**D**) Canonical pathways based on a -log(p-value) greater than 1.5 and an absolute value z-score greater than 2.0. Color by z-score. Blue signifies negative value; orange signifies positive value; and grey signifies no activity pattern. Size is proportional to the number of genes that overlap the pathway. (**E**) Graphical summary for the signaling pathways. Orange and blue indicate activation and inhibition, respectively. Gray dashed lines depict no prediction. (**F**) Schematic cartoon for the relationship between PCSK9-mediated defective efferocytosis and vascular aging. This figure was created with Biorender.com.

We assessed intracellular ROS levels in young (P3) and aged (P15) MAECs isolated from WT and *PCSK9^-/-^* mice using a flow cytometry assay. Whereas there was no significant difference in intracellular ROS level between WT and *PCSK9^-/-^* in young MAECs (**Figure 4A**), ROS levels in aged MAECs were markedly reduced in *PCSK9^-/-^* compared to WT ECs (**Figure 4A**). Our proteomics data showed that Pep2-8 treatment markedly inhibits expression of NADPH oxidase 4 (NOX4), a major source of ROS production,^37^ while Pep2-8 treatment induces expression of antioxidant mediators nuclear respiratory factor 1 (NRF1) and superoxide dismutase 1 (SOD1), suggesting that NOX4, NRF1 and SOD1 are involved in PCSK9-mediated defective efferocytosis and vascular aging (**Figure 4B**). IPA prediction for upstream signaling (**Figure 4C**, **4E** and **4F**) and causal networks (**Figure 4D**) showed that the Mitogen-Activated Protein Kinase (MAPK) pathway, which involves extracellular signal-regulated kinase 1/2 (ERK), protein kinase B (Akt, also called PKB) and P38 MAPK may play a role in PCSK9-mediated defective efferocytosis and vascular aging. Since activation of the MAPK pathway induces both excessive ROS production and the inflammatory response,^38^ we investigated the role of this pathway in PCSK9-mediated defective efferocytosis and vascular aging. Among the top 60 proteins downregulated by Pep2-8 based on activation z-score, we found ERK, Akt and P38 MAPK (**Figure 5A**). Consistently, among the top 60 proteins upregulated by Pep2-8 based on activation z-score (**Figure 5B**), we found SB203580, a selective inhibitor of p38 MAPK, and SB431542, a potent small molecule inhibitor of TGF-β signaling. We also found that Pep2-8 activates several microRNAs, such as miR-124-3p, miR-1-3p and miR-15, providing another novel line of investigation to potentially implicate microRNA regulation in defective efferocytosis. Interestingly, based on activation z-score, we found that Pep2-8 treatment markedly inhibits the NF-κB family (**Figure 5A**), a central mediator of immune and inflammatory responses.^39^ NF-κB induces the expression of various pro-inflammatory genes, including those encoding cytokines and chemokines, and also participates in inflammasome regulation.^39^ Therefore, we examined the proteomics data for inflammatory cytokines or chemokines in the aortic arch from aged mice treated with saline or Pep2-8. As shown in **Figure 5C**, we found that, compared with saline, Pep2-8 significantly downregulated 18 cytokines or chemokines including TNF-α, C-X-C Motif Chemokine Ligand (CXCL) 8/12, and IL-1β, 2, -3, -5, -6, -13, and -20. Pep2-8 also upregulated 10 cytokines and chemokines, such as leukemia inhibitory factor (LIF, an IL-6 class cytokine), nicotinamide phosphoribosyltransferase (NAMPT), ciliary neurotrophic factor (CNTF) and IL-9. Using the IPA system with a threshold of -Log (P-value)>2, **Figure 5D** indicates that 30 canonical pathways were found to be enriched in Pep2-8 vs saline in the aortic arch from the aged mice. Several inflammation pathways such as anti-inflammatory AMP-activated protein kinase (AMPK) signaling and inflammatory eukaryotic initiation factor 2 (eIF2) signaling responded to Pep2-8 treatment. It should be noted that there is only negative regulation overlap in disease-specific pathways and cytokine signaling. The graphical summary for Pep2-8 vs saline in the aged aortic arch is shown in **Figure 5E**. Consistently, we found that Pep2-8 activates Nitric Oxide Synthase Trafficking (NOSTRIN) that is implicated as protective against cardiovascular disease, and inhibits several other pathways regarded as deleterious including synthesis of lipid, systemic autoimmune syndrome and migration of cells. **Figure 5F** summarizes a new possible signaling pathway in PCSK9-mediated vascular aging, whereby PCSK9 induces defective efferocytosis in ECs, subsequently activating MAPK pathways, NF-κB signaling and ROS production. These events induce the release of inflammatory cytokines and chemokines that promotes chronic low-grade inflammation called inflammaging, a known process that aggravates the development of vascular aging.

### Database analysis of PCSK9 in disease and biological functions

**Figure 6:**
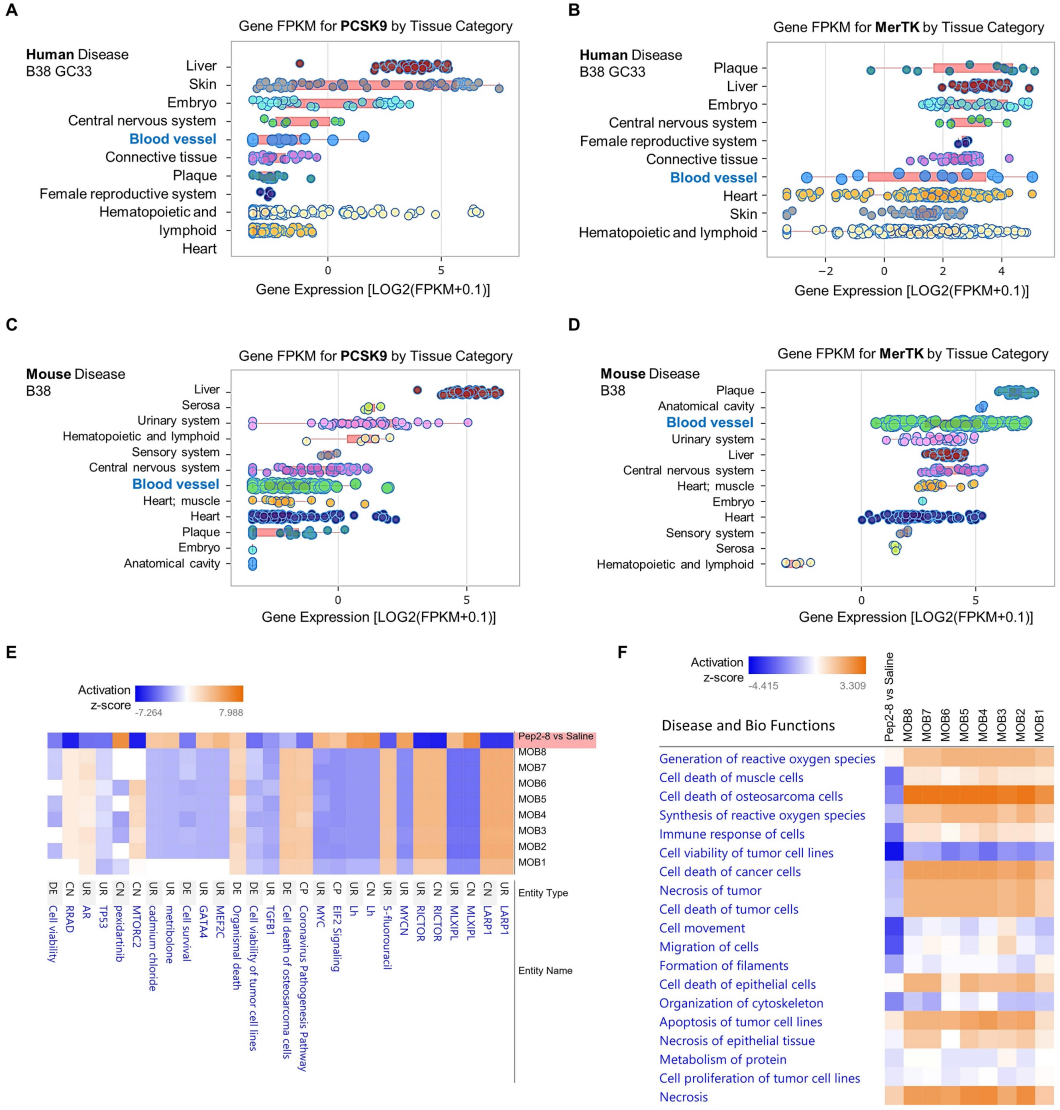
** Pep2-8 treatment and disease and biology functions.** Big data analytics based on gene FPKM for PCSK9 and MerTK in (**A-B**) human disease B38 GC33 and (**C-D**) mouse disease B38. (**E**) Heatmap for representative protein changes between Pep2-8 vs saline in the aortic arch of WT 24-month-old mice and MOB 1-8. (**F**) Heatmap for the disease and biology functions between Pep2-8 vs saline and MOB 1-8.

To further clarify whether PCSK9 may play a broader role in systems biology and disease, we performed data analytics of RNA-seq gene expression with a FDR-adjusted p-value < 0.05 and an absolute fold change > 2 as the gene list analyzed by IPA networks, which were based on Fragments per kilo base of transcript per million mapped fragments (FPKM). In human disease B38 GC33 and mouse disease B38, we found that both PCSK9 and MerTK are highly expressed in blood vessels and plaque, which are closely associated with vascular aging (**Figure 6A-6D**). Obesity is a strong risk factor for the development of aging-mediated CVD because patients with obesity experience CVD events at an earlier age, live with CVD for a greater proportion of their lifetime, and have a shorter average life span than individuals with normal weight.^40^ Therefore, we compared our proteomics results of Pep2-8 treated aortic arch with morbid obesity (MOB) in disease and biological functions. As shown in **Figure 6E**, our proteomics data corroborated the change of several genes at the protein level based on activation z-score. It is noted that compared with eight independent studies of MOB (MOB1-8), Pep2-8 inhibits LARP1 (La Ribonucleoprotein 1, a novel target of the mammalian target of rapamycin complex 1 signaling pathway), EIF2 (eukaryotic Initiation Factor 2) and GATA4 (GATA Binding Protein 4), while it induces RICTOR (Rapamycin-insensitive companion of mammalian target of rapamycin). LARP1, EIF2, GATA4 and RICTOR have been shown to play roles in cell phagocytosis and degradation, indicating their possible role in PCSK9-mediated efferocytosis.^41^-^45^ Collectively, our proteomics analyses in Disease and Bio Functions suggest that Pep2-8 treatment significantly inhibits ROS synthesis and generation, cell death of muscle cells, cell immune response and necrosis (unprogrammed death of cells and living tissue) compared with MOB 1-8 (**Figure 6F**). This analysis also implies that Pep2-8 may inhibit cell death and apoptosis in tumor/cancer cells, indicating that PCSK9 inhibition may provide benefit to vascular aging but potentially predisposing to neoplasms, a possibility that will require careful evaluation.

### Mechanisms of PCSK9-mediated endothelial aging

**Figure 7:**
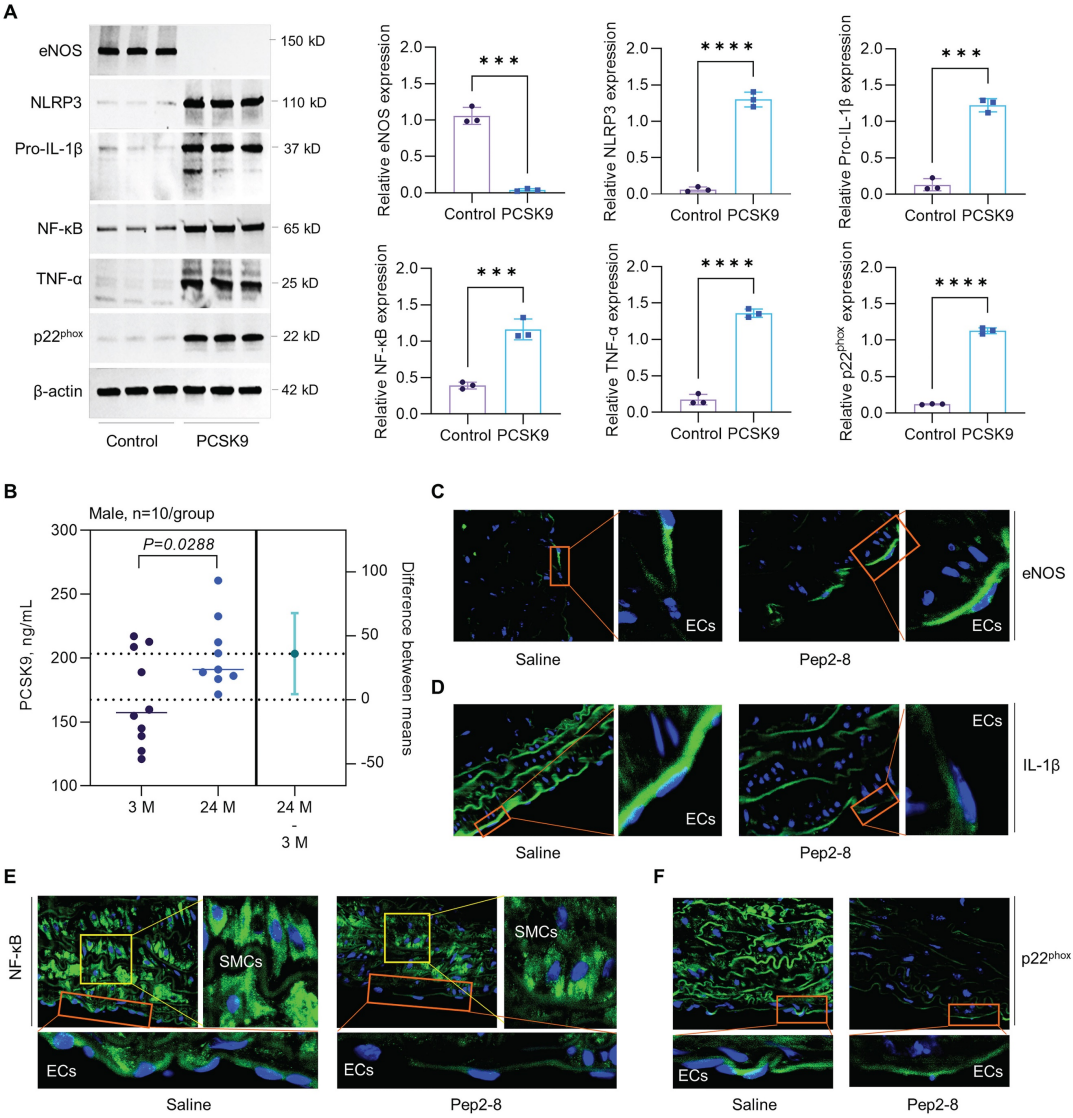
** Effect of PCSK9 and Pep2-8 treatment on aging markers.** (**A**) Western blotting for expression of eNOS, NLRP3, pro-IL-1β, NF-κB, TNF-α and p22^phox^ in HAECs. PCSK9 at 1 µg/mL or PBS control were treated with P15 HAECs for 24 hours. (**B**) ELISA analysis for PCSK9 concentration in serum from young (3-month-old) and aged (24-month-old) WT mice. (**C**) Immunofluorescent staining for eNOS, pro-IL-1β, NF-κB and p22^phox^ in the aortic arch from the aged mice treated with saline or Pep2-8. Statistical analyses were performed with GraphPad Prism 9.4.1 using a two-tailed unpaired t-test. Data represent mean ± SD (n=3 *in vitro* and n=10 *in vivo*). ***P<0.001, ****P< 0.0001.

To clarify whether the increased expression of PSCK9 is the cause or the consequence of aging HAECs, we treated aged HAECs with recombinant PCSK9 and investigated the role of PCSK9 in regulating expression of aging markers, such as endothelial nitric oxide synthase (eNOS), NLR Family Pyrin Domain Containing 3 (NLRP3) inflammasome (NLRP3 and pro-IL-1β), nuclear factor-κB (NF-κB), tumor necrosis factor α (TNF-α), and NADPH oxidase p22^phox^.^46^ eNOS determines endothelial-derived nitric oxide (NO) production and plays an important role in maintaining vascular integrity.^47^ NF-κB is a central mediator for inflammation and immune response.^48^ TNF-α and NLRP3 inflammasome are well known markers for inflammaging, a chronic and sterile low-grade inflammation.^49^ NADPH oxidase is the major source of ROS production and p22^phox^ is an essential component of NADPH oxidase in the vasculature.^50^ Vascular aging is accompanied by the reduced expression of eNOS and enhanced expression of NF-κB, TNF-α, NLRP3 inflammasome and NADPH oxidase.^51^ As shown in **Figure 7A**, our data showed that, compared with control, PCSK9 treatment markedly inhibited expression of eNOS while induced expression of NLRP3, pro-IL-1β, NF-κB, TNF-α and p22^phox^ in P15 HAECs.

Statins is a class of cholesterol-lowering drugs through slowing down cholesterol production in liver.^52^ The role of statins in regulating vascular function has been extensively studied in both vascular ECs and mice that are closely associated with the aging, such as *PCSK9^-/-^*, *LDLr^-/-^* and *ApoE^-/-^* mice. In human vein ECs, simvastatin shows obvious anti-aging effects and atorvastatin inhibits expression of aging markers p53 and p16, and endothelial senescence. Statins also restored vascular endothelial cell function by promoting eNOS expression.^53^,^54^ In *PCSK9^-/-^* mice, lovastatin administration sharply enhanced low density lipoprotein receptor (LDLr) in liver and LDL clearance from plasma.^55^ In *LDLr^-/-^* mice with high fat diet, simvastatin treatment inhibits oxidative stress, artery calcification and expression of TNF-α and subsequent NADPH oxidase subunit.^56^ In *ApoE^-/-^* mice, pitavastatin administration shows lower levels of NADPH oxidases (p47*^phox^*, p47*^phox^*, gp91*^phox^*), toll-like receptor-2/-4, and C-X-C chemokine receptor-4 in aorta.^57^ Another study with *ApoE^-/-^* mice showed that statins promotes eNOS expression and restores vascular endothelial cell function.^58^ In summary, anti-aging effects of stains are mainly based on downregulation of oxidative stress and pro-inflammatory response while upregulation of eNOS expression that leads to enhanced NO bioavailability and improved endothelial function. Therefore, to determine whether the benefit effects of statins are directly related to PSCK9 inhibition, immunofluorescent staining was performed for the aging markers (e.g. eNOS, pro-IL-1β, NF-κB and p22^phox^) in aortic arch from saline or Pep2-8 treated mice. First, our data by ELISA showed that PCSK9 concentration in serum was significantly increased in the aged mice compared with young mice (**Figure 7B**). Second, our data by immunofluorescent staining showed that, compared with saline treated group, Pep2-8 administration upregulated expression of eNOS while downregulated expression of pro-IL-1β, NF-κB and p22^phox^, indicating the similar anti-aging effects between statins and PCSK9 inhibition (**Figure 7C-F**). Besides eNOS that is predominantly expressed in endothelium (**Figure 7C**), pro-IL-1β, NF-κB and p22^phox^ are extensively expressed in the whole aorta tissues (**Figure 7D-F**). Of note, NF-κB is highly expressed in smooth muscle cells (SMCs) compared with ECs. Pep2-8 treatment significantly inhibited NF-κB expression in SMCs, providing the novel ideas for our further study focusing on PCSK9 and SMC biology in vascular aging.

## Discussion

The vascular aging component of cardiovascular disease is partly a consequence of EC senescence,^1^ which is accompanied by a chronic inflammatory response and excessive ROS production.^59^ The mechanisms that mediate EC senescence are not well understood. Our data based on molecular biology and proteomics reveal an inverse relationship between PCSK9 and EC efferocytosis that may contribute to vascular aging. We found that PCSK9 increases in aortic endothelial cells during aging, which is associated with a loss of the efferocytosis receptor MerTK. Recombinant PCSK9 downregulates MerTK and induces defective efferocytosis whereas PCSK9 inhibition restores efferocytosis and MerTK in both ECs ex-vivo and in aortic endothelium of aged mice. Furthermore, we showed that excessive ROS production and chronic inflammation as features of vascular aging are mitigated in aorta of PCSK9^-/-^ mice. Our observations improve the current understanding of the pathogenesis of vascular aging and indicate that targeting the PCSK9/MerTK pathway might restrain the development of vascular aging.

ECs are the unique barrier to inflammation and only activated ECs express the directing signals that control access of immune cells to underlying tissues.^4^ Recent studies show that PCSK9 is highly expressed in vascular cells, including EC, smooth muscle cells and macrophages.^7^,^8^,^16^ PCSK9 induces ROS production via NOX2 and NOX4 in ECs.^7^,^8^ Silencing of PCSK9 decreases expression of endothelial CX3CL1 and CXCL16, and reduces secretion of cytokines such as TNF-α, IL-1β, and IL-18 and chemokines such as chemotaxis of neutrophils (IL-8/CXCL8) and eosinophils (eotaxin-2/CCL24).^60^ Consistent with published data,ref our flow cytometry data demonstrate that *PCSK9^-/-^* significantly inhibits ROS production in aged ECs. Our proteomics data further show that inhibition of PCSK9 by Pep2-8 treatment markedly regulates redox signaling including downregulation of NOX4 and upregulation of antioxidant NRF1 and SOD1 in aorta of aged mice, and additionally influences a variety of inflammatory cytokines such as CXCL8/12, IL-1β and LIF. Although all of these processes are involved in the development of EC senescence, we do not fully understand the complex signaling pathways by which PCSK9 promotes senescence in the vascular endothelium.

A recent study showed that MerTK, a critical receptor of efferocytosis, is highly expressed in ECs, indicating the potential for ECs to conduct efferocytosis.^28^ Oxidative stress and inflammatory factors such as ox-LDL and LPS drive defective efferocytosis, leading to secretion of pro-inflammatory cytokines such as IL-1β and TNF-α.^13^ Therefore, we posited that MerTK plays a role in PCSK9-mediated defective efferocytosis and subsequent vascular aging. As expected, our data showed that recombinant PCSK9 treatment inhibits MerTK expression particularly in aged ECs, whereas PCSK9 inhibition restores MerTK expression in both ECs ex-vivo and intact endothelium. In-vivo deletion of PCSK9 in *PCSK9^-/-^* mice also significantly reduced SA-β-gal activity in aged ECs and the aortic arch, whereas treatment of aged mice with recombinant PCSK9 accentuated SA-β-gal activity in this vessel that is prone to disturbed flow accompanied by endothelial dysfunction. Collectively, these findings provide strong rationale for considering PCSK9 as a negative regulator of MerTK and efferocytosis in ECs and argue that repurposing clinically-used PCSK9 inhibitors may have value for the treatment of vascular diseases associated with aging and defective efferocytosis.

We performed a series of proteomic analyses using aorta from Pep2-8 treated mice to find clues to unique pathways through which PCSK9 may signal to exert its anti-efferocytotic effect, and conversely, gain insight into mechanisms by which PCSK9 inhibitors confer protection from defective efferocytosis and vascular aging. Age-related and tissue-specific changes in lipid composition contribute to the aging process. Our proteomic data provided evidence that Pep2-8 regulates lipid levels by decreasing LDL and increasing HDL in the aged mice. Activation of MAPK pathways promotes EC senescence and vascular aging. In addition, activation of MAPK and NF-κB induces inflammation and defective efferocytosis. Our proteomic study shows that Pep2-8 treatment significantly inhibits the MAPK pathway (ERK, Akt and P38) and NF-κB signaling, leading to downregulation of a variety of pro-inflammatory cytokines and chemokines. Collectively, our results from canonical pathways and graphical summary provide strong evidence for the protective role of PCSK9 inhibition in vascular aging. Our big data analytics indicate that Pep2-8 treatment blocks the synthesis and generation of ROS and regulates several inflammatory signals. These data infer that these pathways represent mechanisms by which PCSK9 and MerTK mediate defective efferocytosis as a contributor to EC senescence and vascular aging.

Previously, we reported that pretreatment with NADPH oxidase inhibitors diphenylene-iodonium chloride (DPI) and apocynin markedly inhibited ROS production and subsequent PCSK9 expression in HAECs.^7^,^8^ Further study showed that recombinant PCSK9 treatment induced while PCSK9 knockdown significantly inhibited ROS production in HAECs, indicating a bidirectional interaction between PCSK9 and ROS production.^7^,^8^ Consistently, our current study showed that PCSK9 and ROS production increase in aged HAECs and PCSK9 plays a key role in regulating ROS production. In addition, the hallmark of the ageing process is the increase of pro-inflammatory markers in blood and tissues, while pro-inflammatory factors (e.g. LPS, IL-1β and oxidized LDL) induce PCSK9 secretion in a variety of vascular cells.^51^,^61^ Based on these findings, it is reasonable to conclude that, increased ROS production and pro-inflammatory response, are the potential mechanisms for increasing PCSK9 expression in aging ECs. It exits an increasing imbalance shown as elevated oxidative stress and reduced eNOS-mediated NO production during aging. Of note, our data showed that recombinant PCSK9 treatment almost blocked total expression of eNOS in aged HAECs, indicating that eNOS is very sensitive to PCSK9 treatment. Immunofluorescent staining for eNOS expression in aortic arch showed that PCSK9 inhibition restores eNOS expression from the aged mice. Interestingly, we also found the similar anti-aging effects between statins and PCSK9 inhibition, such as enhanced expression of eNOS and inhibited expression of oxidative stress and pro-inflammatory response.

In conclusion, we provide evidence for a relationship between PCSK9, MerTK and efferocytosis in ECs and its implications in vascular aging. ECs have a high ability to perform efferocytosis when young, but this ability decreases with increasing age. PCSK9 signaling is central to the response of aged ECs in redox biology and secretion of cytokines or chemokines and PCSK9 inhibition causes complex changes in expression of proteins involved in LDL and HDL-C biology, ROS production, and inflammatory responses. Functional EC efferocytosis protects against pathogenic changes that predispose arteries to senescence and our data provide important translational clues that PCSK9 may represent a strong therapeutic target for the treatment of vascular aging.

## References

[1] Donato AJ, Machin DR, Lesniewski LA (2018). Mechanisms of dysfunction in the aging vasculature and role in age-related disease. Circulation Res.

[2] Jia G, Aroor AR, Jia C, Sowers JR (2019). Endothelial cell senescence in aging-related vascular dysfunction. Biochimica et Biophysica Acta (BBA)-Molecular Basis of Disease.

[3] Tejero J, Shiva S, Gladwin MT (2019). Sources of vascular nitric oxide and reactive oxygen species and their regulation. Physiol Reviews.

[4] Shao Y, Saredy J, Yang WY, Sun Y, Lu Y, Saaoud F (2020). Vascular endothelial cells and innate immunity. Artherio Thromb Vas Bio.

[5] Rea IM, Gibson DS, McGilligan V, McNerlan SE, Alexander HD, Ross OA (2018). Age and age-related diseases: role of inflammation triggers and cytokines. Front. Immun.

[6] Surdo PL, Bottomley MJ, Calzetta A, Settembre EC, Cirillo A, Pandit S (2011). Mechanistic implications for LDL receptor degradation from the PCSK9/LDLR structure at neutral pH. EMBO reports.

[7] Ding Z, Liu S, Wang X, Deng X, Fan Y, Shahanawaz J (2015). Cross-talk between LOX-1 and PCSK9 in vascular tissues. Cardiovas Res.

[8] Ding Z, Liu S, Wang X, Deng X, Fan Y, Sun C (2015). Hemodynamic shear stress via ROS modulates PCSK9 expression in human vascular endothelial and smooth muscle cells and along the mouse aorta. Antioxi Redox Sig.

[9] Momtazi-Borojeni AA, Sabouri-Rad S, Gotto Jr AM, Pirro M, Banach M, Awan Z (2019). PCSK9 and inflammation: a review of experimental and clinical evidence. Europ Heart J-Cardiovas Pharm.

[10] Rea IM, Gibson DS, McGilligan V, McNerlan SE, Alexander HD, Ross OA Age and age-related diseases: role of inflammation triggers and cytokines. Front Immunol. 2018: 586.

[11] Ameisen JC (2002). On the origin, evolution, and nature of programmed cell death: a timeline of four billion years. Cell Death Differ.

[12] Elliott MR, Koster KM, Murphy PS (2017). Efferocytosis signaling in the regulation of macrophage inflammatory responses. J Immunology.

[13] Yurdagul Jr A, Doran AC, Cai B, Fredman G, Tabas IA (2018). Mechanisms and consequences of defective efferocytosis in atherosclerosis. Front Cardiovas Med.

[14] De Maeyer RP, van de Merwe RC, Louie R, Bracken OV, Devine OP, Goldstein DR (2020). Blocking elevated p38 MAPK restores efferocytosis and inflammatory resolution in the elderly. Nat Immun.

[15] Tower J (2015). Programmed cell death in aging. Ageing Res Reviews.

[16] Ding Z, Wang X, Liu S, Zhou S, Kore RA, Mu S (2020). NLRP3 inflammasome via IL-1β regulates PCSK9 secretion. Theranostics.

[17] Cai B, Thorp EB, Doran AC, Sansbury BE, Daemen MJ, Dorweiler B (2017). MerTK receptor cleavage promotes plaque necrosis and defective resolution in atherosclerosis. J Clin Investigation.

[18] Cai B, Kasikara C, Doran AC, Ramakrishnan R, Birge RB, Tabas I (2018). MerTK signaling in macrophages promotes the synthesis of inflammation resolution mediators by suppressing CaMKII activity. Science Signaling.

[19] Wang JM, Chen AF, Zhang K (2016). Isolation and primary culture of mouse aortic endothelial cells. JoVE.

[20] Liao Y, Smyth GK, Shi W (2014). feature Counts: an efficient general purpose program for assigning sequence reads to genomic features. Bioinforma Oxf Engl.

[21] Robinson MD, Oshlack A (2010). A scaling normalization method for differential expression analysis of RNA-seq data. Genome Biol.

[22] Ritchie ME, Phipson B, Wu D, Hu Y, Law CW, Shi W (2015). Limma powers differential expression analyses for RNA-sequencing and microarray studies. Nucleic Acids Research.

[23] Searle BC, Pino LK, Egertson JD, Ting YS, Lawrence RT, MacLean BX (2018). Chromatogram libraries improve peptide detection and quantification by data independent acquisition mass spectrometry. Nat Commun.

[24] Graw S, Tang J, Zafar MK, Byrd AK, Bolden C, Peterson EC, Byrum SD (2020). proteiNorm - A user-friendly tool for normalization and analysis of TMT and label-free protein quantification. ACS Omega.

[25] Zhou X, Lindsay H, Robinson MD (2014). Robustly detecting differential expression in RNA sequencing data using observation weights. Nucleic Acids Research.

[26] Proto JD, Doran AC, Gusarova G, Yurdagul Jr A, Sozen E, Subramanian M (2018). Regulatory T cells promote macrophage efferocytosis during inflammation resolution. Immunity.

[27] De Couto G, Jaghatspanyan E, DeBerge M, Liu W, Luther K, Wang Y (2019). Mechanism of enhanced MerTK-dependent macrophage efferocytosis by extracellular vesicles. Arterio Thromb. Vas. Bio.

[28] Li Y, Wittchen ES, Monaghan-Benson E, Hahn C, Earp HS, Doerschuk CM (2019). The role of endothelial MERTK during the inflammatory response in lungs. PLoS One.

[29] Zhang J, Tecson KM, Rocha NA, McCullough PA (2018). Usefulness of alirocumab and evolocumab for the treatment of patients with diabetic dyslipidemia. Proc (Bayl Univ Med Cent).

[30] Zhang Y, Eigenbrot C, Zhou L, Shia S, Li W, Quan C (2014). Identification of a small peptide that inhibits PCSK9 protein binding to the low density lipoprotein receptor. J Bio. Chem.

[31] Loyer X, Potteaux S, Vion AC, Guérin CL, Boulkroun S, Rautou PE (2014). Inhibition of microRNA-92a prevents endothelial dysfunction and atherosclerosis in mice. Circulation Res.

[32] Schubert SY, Benarroch A, Monter-Solans J, Edelman ER (2011). Primary monocytes regulate endothelial cell survival through secretion of angiopoietin-1 and activation of endothelial Tie2. Arterio. Thromb. Vas. Bio.

[33] Sabatine MS (2019). PCSK9 inhibitors: clinical evidence and implementation. Nat Reviews Cardio.

[34] Martínez-Zamudio RI, Dewald HK, Vasilopoulos T, Gittens-Williams L, Fitzgerald-Bocarsly P, Herbig U (2021). Senescence-associated β-galactosidase reveals the abundance of senescent CD8+ T cells in aging humans. Aging Cell.

[35] Rosenson RS, Hegele RA, Koenig W (2019). Cholesterol-lowering agents: PCSK9 inhibitors today and tomorrow. Circulation Res.

[36] Franceschi C, Campisi J (2014). Chronic inflammation (inflammaging) and its potential contribution to age-associated diseases. J Geront Series A: Biomedical Sciences and Medical Sciences.

[37] Canugovi C, Stevenson MD, Vendrov AE, Hayami T, Robidoux J, Xiao H (2019). Increased mitochondrial NADPH oxidase 4 (NOX4) expression in aging is a causative factor in aortic stiffening. Redox Bio.

[38] Meng Z, Yan C, Deng Q, Gao DF, Niu XL (2013). Curcumin inhibits LPS-induced inflammation in rat vascular smooth muscle cells *in vitro* via ROS-relative TLR4-MAPK/NF-κB pathways. Acta Pharmacologica Sinica.

[39] Hayden MS, Ghosh S (2011). NF-κB in immunobiology. Cell Res.

[40] Miller M, Stone NJ, Ballantyne C, Bittner V, Criqui MH, Ginsberg HN (2011). Triglycerides and cardiovascular disease: a scientific statement from the American Heart Association. Circulation.

[41] Uribe-Querol E, Rosales C (2020). Phagocytosis: our current understanding of a universal biological process. Front Immun.

[42] Hong S, Freeberg MA, Han T, Kamath A, Yao Y, Fukuda T (2017). LARP1 functions as a molecular switch for mTORC1-mediated translation of an essential class of mRNAs. Elife.

[43] Shrestha N, Bahnan W, Wiley DJ, Barber G, Fields KA, Schesser K (2012). Eukaryotic initiation factor 2 (eIF2) signaling regulates proinflammatory cytokine expression and bacterial invasion. J Biol Chem.

[44] Huerta M, Reyes L, García-Rivera G, Bañuelos C, Betanzos A, Díaz-Hernández M (2020). A noncanonical GATA transcription factor of Entamoeba histolytica modulates genes involved in phagocytosis. Molec Microbiol.

[45] Oh MH, Collins SL, Sun IH, Tam AJ, Patel CH, Arwood ML (2017). mTORC2 signaling selectively regulates the generation and function of tissue-resident peritoneal macrophages. Cell Reports.

[46] Xia X, Chen W, McDermott J, Han JD (2017). Molecular and phenotypic biomarkers of aging. F1000Research.

[47] Tran N, Garcia T, Aniqa M, Ali S, Ally A, Nauli SM (2022). Endothelial nitric oxide synthase (eNOS) and the cardiovascular system: In physiology and in disease states. Am J Biomed Sci Res.

[48] Baker RG, Hayden MS, Ghosh S (2011). NF-κB, inflammation, and metabolic disease. Cell Metab.

[49] Franceschi C, Garagnani P, Vitale G, Capri M, Salvioli S (2017). Inflammaging and 'Garb-aging'. Trends Endocrinol Metab.

[50] Camargo LL, Montezano AC, Hussain M, Wang Y, Zou Z, Rios FJ, Neves KB, Alves-Lopes R, Awan FR, Guzik TJ, Jensen T (2022). Central role of c-Src in NOX5-mediated redox signalling in vascular smooth muscle cells in human hypertension. Cardiovas Res.

[51] Ungvari Z, Tarantini S, Donato AJ, Galvan V, Csiszar A (2018). Mechanisms of vascular aging. Circ Res.

[52] Yu D, Liao JK (2022). Emerging views of statin pleiotropy and cholesterol lowering. Cardiovas Res.

[53] Lei J, Gu X, Ye Z, Shi J, Zheng X (2014). Antiaging effects of simvastatin on vascular endothelial cells. Clin Appl Thromb Hemost.

[54] Zhang JJ, Zhang YZ, Peng JJ, Li NS, Xiong XM, Ma QL (2018). Atorvastatin exerts inhibitory effect on endothelial senescence in hyperlipidemic rats through a mechanism involving down-regulation of miR-21-5p/203a-3p. Mech Ageing Dev.

[55] Rashid S, Curtis DE, Garuti R, Anderson NN, Bashmakov Y, Ho YK, Hammer RE, Moon YA, Horton JD (2005). Decreased plasma cholesterol and hypersensitivity to statins in mice lacking Pcsk9. Proc Natl Acad Sci USA.

[56] Lin CP, Huang PH, Lai CF, Chen JW, Lin SJ, Chen JS (2015). Simvastatin attenuates oxidative stress, NF-κB activation, and artery calcification in LDLR^-/-^ mice fed with high fat diet via down-regulation of tumor necrosis factor-α and TNF receptor 1. PloS one.

[57] Lei Y, Cui Q, Yang G, Piao L, Inoue A, Wu H, Li X, Kuzuya M, Cheng XW (2021). Statins mitigate stress-related vascular aging and atherosclerosis in apoE-deficient mice fed high fat-diet: the role of glucagon-like peptide-1/adiponectin axis. Front Cell Dev Biol.

[58] Motoji Y, Fukazawa R, Matsui R, Abe Y, Uehara I, Watanabe M, Hashimoto Y, Miyagi Y, Nagi-Miura N, Tanaka N, Ishii Y (2022). Statins Show Anti-Atherosclerotic Effects by improving endothelial cell function in a Kawasaki Disease-like vasculitis mouse model. Int J Mol Sci.

[59] Bhattacharyya A, Chattopadhyay R, Mitra S, Crowe SE (2014). Oxidative stress: an essential factor in the pathogenesis of gastrointestinal mucosal diseases. Physiol Reviews.

[60] Marques P, Domingo E, Rubio A, Martinez-Hervás S, Ascaso JF, Piqueras L (2022). Beneficial effects of PCSK9 inhibition with alirocumab in familial hypercholesterolemia involve modulation of new immune players. Biomed Pharma.

[61] Barale C, Melchionda E, Morotti A, Russo I (2022). PCSK9 biology and its role in atherothrombosis. Int J Mol Sci.

